# Dr. Hall, on Hydrophobia

**Published:** 1806-05

**Authors:** R. Hall

**Affiliations:** Clement's Inn


### *To the Editors of the Medical and Physical Journal.*

GENTLEMEN,

THOUGH there is scarcely any disease that has more engaged the attention of physicians than rabid hydrophobia, yet we have still to lament, that on no subject whatever have medical learning, skill and ingenuity, been exerted with effects more disproportionate; for it is undeniable that notwithstanding all the remedies hitherto proposed, either as prophylactic or curative, the malady still continues to occur, and to prove as intractable as at any former period.*

Many cases have, it is true, at different times, been published, in which cures are said to have been performed; but if these statements be examined with attention, we shall, for the most part, see reason to suppose, that the authors of them had mistaken some other disease for hydrophobia.

Of this kind, according to our apprehension, is the recent account given by Professor Rossi, in his reports of the efficacy of galvanism in two cases of supposed rabies.† The same remark, it appears to us, is equally applicable to an account of a case of hydrophobia, communicated by Dr. Burton, of Bent, in Virginia, to Dr. Rush of Philadelphia, and published in the 78th Number of the Medical and Physical Journal. It is therein asserted, that the disease, in this instance, was effectually combated by copious bleeding and mercury.

---

* When treating, in the 73d Number of the Medical and Physical Journal, of the external means employed in the way of prevention against hydrophobia, we took occasion to reprobate, as an error fraught with the most dangerous consequences, the conduct of those practitioners who recommend, in the most unqualified manner, particular modes of practice to the exclusion of the only certain preventive of this dreadful malady, the excision or destruction of the bitten part.

The arguments contained in that paper apply, it must be admitted, with equal force to the different internal remedies, hitherto so frequently extolled as specifics in preventing this formidable disorder, since their inefficacy is fully proved by the appearance of the disease after they have been administered with the greatest care. Hence too much pains cannot be taken to correct the existing prejudices in their favour, and to inculcate, on every occasion, the danger that may ensue from allowing such an ill-grounded confidence to supersede the timely and effectual removal of the injured part.

† Medical and Physical Journal, No. 67.

After the strictures by Dr. Bardsley on Professor Rossi's Reports, (Med. and Phys. Journal, No. 69) we deem it wholly unnecessary to enter into any farther investigation on that subject, and at present intend to confine ourselves to the consideration of the case related by Dr. Burton, on which, we trust, we may be permitted to offer such observations as a candid and impartial review of the subject shall appear to us to demand.

In the case to which we here allude, we must first observe, that no proof whatever is adduced of the absolute madness of the animal, when the patient sustained the injury; it is merely mentioned, that he had been bitten by a dog, which his friends suspected to be mad; but on what this conjecture was founded, we are left in complete ignorance. It is a fact verified by repeated observation, that scarcely one animal out of forty, supposed to be rabid, is really so; which affords an unequivocal proof how little reliance can be placed on the testimony of patients or their friends on such occasions. Unless, therefore, proper measures be pursued, so as to ascertain, beyond contradiction, the madness of the dog, doubts must always arise, as in the present case, how far the symptoms that may supervene, are imputable to the bite of a rabid animal.

It has also in general been too rashly inferred, that when persons are injured by animals actually rabid, hydrophobia must necessarily occur; but this by no means follows, for it is an indisputable fact, that out of twenty persons so injured, nineteen on an average escape without becoming affected by the disease.

We are not told in this case, that any mode of prevention whatever was employed, so that the patient was probably left to indulge his apprehensions of the consequences of the accident; which appears, from the whole tenor of the account, to have made a deep impression on his mind.

The bite, we are informed, was inflicted on one of the lower extremities, but nothing is said respecting the state of the bitten part; whether it looked red, assumed an inflamed appearance, or underwent any change previous to the attack, or during the continuance of the complaint. We are indeed told, that the patient, after his convalescence, informed Dr. Burton, in answer to some inquiries made by him to this purpose, that he had felt a slight pain attended with an itching and uneasiness in his groin, for several days previous to his illness; but when it is considered sidered that he made no complaint of this kind, either before the attack or during the intervals of the paroxysms, as they are termed, nor was ever observed putting his hand to the bitten part, which is not unusual with patients labouring under this dreadful malady, very little importance, it should seem, can be attached to this account; more particularly, when the evident prepossession of the patient, as to the nature of his disease, is taken into consideration.

The length of time which intervened between the infliction of the bite and the accession of the complaint, in the present instance, is stated to have been twenty-four days, which is a shorter interval than usual; for, in well marked cases of hydrophobia, the disease seldom makes its appearance sooner than five or six weeks after the accident. As, however, much may depend on the susceptibility of the system at the time the injury is sustained, the virulence of the poison, and various other circumstances, considerable latitude must, it is obvious, be allowed as to the period of the attack. But when, along with the absence of most, if not all, of the diagnostic symptoms of true rabies, any unusual deviation occurs in this respect, as was the case with Doctor Burton's patient, it affords additional ground to suspect, that the symptoms may proceed from the operation of a cause wholly unconnected with the application of hydrophobic virus.

Several days previous to the appearance of the disease Dr. Burton, we are told, was informed that the patient had been dull and solitary, and that his friends, a few minutes before the attack, had found him two hundred yards from the house, apparently in a deep study. This fact, while it tends to prove that the mind of the patient was from the first in a very disturbed state, evidently militates against the supposition of rabid hydrophobia; for it is a leading symptom of this disorder, that the patient cannot bear exposure even o the air of a common room, without experiencing great uneasiness.*

The symptoms enumerated by Dr. Burton, when he first visited the patient, were, a dull pain of the head, watery

* This extreme aversion to the contact or application of cold air or moisture did not escape the notice of some of the more early writers on this subject. Among other symptoms, Cælius Aurelianus mentions it as one of the signs of approaching hydrophobia: His words are, "Insueta etiam querela aeris, tanquam austrini, quamvis serena fuerit quies; item difficilis toleratio, atque tædium, &. recusatio imbrium."

eyes, dull aspect, stricture, and heaviness at the breast, and a high fever; all which are common to a variety of disorders, and display nothing characteristic of rabid hydrophobia. We are indeed immediately afterwards told, that the patient rejected every thing aqueous; and in a subsequent letter to Dr. Rush, who had requested more precise information respecting several particulars, we are given to understand, that he refused to swallow liquids, and that the sight of water threw him into a convulsive agitation. But, admitting this statement to be correct, it does not thence follow that the malady, under which the patient laboured, was rabid hydrophobia; for it is well known to every intelligent practitioner, that such symptoms are frequently concomitants of other diseases. Innumerable instances of this kind might be adduced; but as the fact is too generally known to render that necessary, we shall only mention a few, which appear to us more particularly remarkable.

Boerhaave relates a case, in which the difficulty of drinking, and consequent dread of water, accompanied an acute fever, occasioned by excessive heat and fatigue, superadded to the intemperate use of ardent spirits.* A very singular case is recorded in the Edinburgh Medical Essays, vol. i. in which hydrophobia appears to have depended on an inflammatory state of the stomach, without the most distant suspicion of the patient having been injured by any rabid animal. Whether this might be in reality a case of gastritis, appears somewhat problematical, for few of the diagnostic symptoms of that affection were present; but be this as it may, the patient frequently ejected saliva from his mouth, and experienced all the horror and anguish at the sight of water, which are so conspicuous in persons labouring under genuine hydrophobia. A case of hydrophobia supervening to a phrenitic attack is related by Dr. Raymond in the Appendix to the 5th vol. of the Medical Observations and Inquiries. We ourselves once witnessed this occurrence, during a phrenitic delirium, in a patient who rejected water and other liquids with a kind of horror, as often as they were presented to him. The late fever at Leghorn, according to Dr. Palloni, frequently manifested itself by the appearance of hydrophobic and other extraordinary symptoms. While these and other facts serve to explain many of the various and contradictory

---

* Van Swieten Comment. Tom. iii.

tradictory reports respecting the effects of different remedies in rabies, they evince the necessity of a clear and accurate discrimination between it and other apparently similar diseases; since there seems but too much reason to suppose, that various affections of an essentially different nature have frequently been confounded with it, merely because the patient had felt an aversion to water at some period of the disorder.*

Whoever attentively examines all the particulars of the case related by Dr. Burton, must, we think, be convinced that he has fallen into this error, and mistaken an affection of a very different nature for genuine hydrophobia. From the very commencement of the disease, the delirium of the patient appears to have been attended with furor; he raved almost incessantly, and bit at every thing indiscriminately within his reach; he was bled; blisters were applied; and in every respect he was treated on the antiphlogistic plan. But although, by these means, the violence of the complaint was somewhat abated, it recurred with increased aggravation on the morning of the third day from the attack, when we are told, he was found biting the bed-clothes; that his countenance was maniacal, his pulse synocha, with stricture of the breast, difficult deglutition, laborious breathing, and a discharge of saliva. All these alarming symptoms were, however, at last, effectually subdued by copious and repeated bleeding: mercurial frictions were also resorted to; but the complaint had evidently yielded before they were employed.

* What has proved the most frequent source of this error, we have every reason to suppose, is the application of the term hydrophobia to designate the disease itself: a term, which taken in its literal and etymological sense, is merely descriptive of a single symptom, which, so far from being confined to true rabies, is not an unusual concomitant of various and very opposite affections. With a view to obviate this source of error, various terms have been proposed by different writers on rabies as a substitute for that of hydrophobia, but all of them appear to be subject to more or less objection. It would, indeed, be difficult to find a single term sufficiently comprehensive fully to express that state of the system which is induced by the action of the rabid virus. Hygrophobia (Υγροφοϐία), which the Greeks themselves employed, is perhaps less exceptionable than most others, in as much as it is more extensive in its import, and expresses not only an antipathy to water, but that preter-natural aversion to all fluids, whether in an aerial or more palpable form, which is so characteristic of true rabies; the latter term, it must be confessed, is somewhat less euphonous than the former, but if it be preferable in other respects, that is a consideration of very inferior importance.

During the short space of four days, the patient lost 138 ounces of blood.

From the history of this case it appears sufficiently obvious, that violent inflammatory action and acute pyrexia prevailed throughout the whole progress, and constituted the principal part of the disease: circumstances which are never observable in well defined cases of hydrophobia.—On a careful review of the whole concourse of the symptoms, as detailed by Dr. Burton, we are of opinion, that they must appear to every impartial observer far more characteristic of a phrenitic attack than of true rabies; for in phrenitis, the raving is almost incessant, with few intervals of reason; whereas in rabies, the paroxysms are short and sudden, and the patient is seldom bereft of all consciousness and perception of what is passing around him. In phrenitis, moreover, the delirious furor early manifests itself, and the patients are generally outrageous; but in rabies, on the contrary, timidity is a prevailing feature, and the patient seldom attempts to injure any of the byestanders, or becomes outrageous, till towards the very close of the malady. The only symptoms in which the disease under consideration bore the slightest resemblance to rabies, were, the patients refusal to swallow liquids, and the convulsive agitation he is said to have experienced at the sight of water. Now these symptoms, as already shown, are no uncommon occurrence in phrenitic, as well as in maniacal affections. In phrenitis, besides, it is well known, that the mind frequently recurs to objects which had before made a deep impression on it. That this was the case in the present instance, is clearly evinced by the patient continually warning people to keep at a distance from him, for he had been bitten by a mad dog. We are fully aware that the desiderium aut cupiditas mordendi is given as a part of the character of this disease by several Nosologists, and that many writers consider it as a proper and inseparable sign of rabies; but whatever stress may be attached to this circumstance by some authors, it can only be considered as a symptom of delirium which is common to several disorders, and therefore furnishes no proof of the patient having been affected with canine madness.

With regard to the discharge of saliva in this instance, it seems altogether to have arisen from impeded deglutition, and the convulsive action of the muscles of the throat and fauces, and is well known to be a frequent occurrence in epileptic as well as in manaical and phrenitic affections; affections; to the last of which the disease in question appears to have had a more striking resemblance, not only in the mode of its attack, but in its subsequent symptoms, than to the peculiar malady resulting from the bite of a rabid animal. It was not marked by any of the leading symptoms of hydrophobia rabiosa; such as an aversion to noise and cold air, to the sound or contact of water and other liquids, to any violent contractions of the lower jaw, any strictures about the throat, with occasional apprehensions of immediate suffocation or strangulation, any violent efforts to disengage from the mouth a thick and viscid saliva, any change of voice, incessant restlessness, and a marked aversion to a recumbent posture. Lastly, it not only differed from this dreadful disorder by the absence of all these symptoms, but in its termination, which was so unlike that of true rabies, hitherto deemed incurable, that it yielded in less than a week to copious and repeated bleedings, and the employment of other remedies:—means which, in the hands of other practitioners, have uniformly proved inefficient. On the whole, so far as we can form any judgment from a comprehensive view of the present case, there seems, we think, every reason to conclude that, whatever might be the disease under which the patient laboured, it was not genuine hydrophobia.

I am, &c.

*Clement's Inn,*

*March* 28, 1806. R. HALL.

